# Routing of Electric Vehicles With Intermediary Charging Stations: A Reinforcement Learning Approach

**DOI:** 10.3389/fdata.2021.586481

**Published:** 2021-05-26

**Authors:** Marina Dorokhova, Christophe Ballif, Nicolas Wyrsch

**Affiliations:** Photovoltaics and Thin Film Electronics Laboratory (PV-Lab), Institute of Microengineering (IMT), École Polytechnique Fédérale de Lausanne (EPFL), Neuchâtel, Switzerland

**Keywords:** electric vehicle, energy management, Q-learning, reinforcement learning, vehicle routing

## Abstract

In the past few years, the importance of electric mobility has increased in response to growing concerns about climate change. However, limited cruising range and sparse charging infrastructure could restrain a massive deployment of electric vehicles (EVs). To mitigate the problem, the need for optimal route planning algorithms emerged. In this paper, we propose a mathematical formulation of the EV-specific routing problem in a graph-theoretical context, which incorporates the ability of EVs to recuperate energy. Furthermore, we consider a possibility to recharge on the way using intermediary charging stations. As a possible solution method, we present an off-policy model-free reinforcement learning approach that aims to generate energy feasible paths for EV from source to target. The algorithm was implemented and tested on a case study of a road network in Switzerland. The training procedure requires low computing and memory demands and is suitable for online applications. The results achieved demonstrate the algorithm’s capability to take recharging decisions and produce desired energy feasible paths.

## 1 Introduction

The importance of electric vehicles (EVs) has increased steadily over the past few years with growing concerns about climate change, volatile prices of fossil fuels and energy dependencies between countries. The transportation sector accounts for 27% of global greenhouse gas emissions in the EU, 72% of which are contributed by road transport (European Environmental Agency, 2019). Therefore, switching to electric mobility is seen as a primary mean of reaching emissions’ reduction targets. Although the EV deployment grows fast around the world (+40% in 2019) with Europe accounting for 24% of the global fleet, specific barriers for a massive uptake of EVs still exist (International Energy Agency, 2020). Researchers in (Noel et al., 2020) identify technical, economic, social and political barriers of EVs’ broad adoption with limited cruising range and sparse charging infrastructure prevailing at present. These barriers are in the essence of the “range anxiety problem” defined as a fear that an EV will not have sufficient charge to reach its destination. However, optimal EV route planning together with higher-range EVs entering the market can mitigate this problem.

Route planning strategies have been widely researched for conventional fossil-fuel vehicles. However, to solve the same problem for EVs, one should consider specific characteristics of this technology, such as limited battery capacity and ability to recuperate energy. Moreover, inadequate charging infrastructure and long charging times call for selective choice of charging stations. Significant factors influencing this choice include the price of electricity, expected charging power, distance from EV to charging station, the current state of charge, expected waiting and charging times, and incentives from electricity providers. Another difficulty in route planning for EVs lies in the choice of the optimization goal. Conventional routing algorithms, such as Dijkstra (Dijkstra, 1959), yield either the least travelled time or distance. However, none of these options guarantees the generated route’s energy feasibility. Therefore, a need for EV-specific routing algorithms that strive for energy efficiency emerged.

The algorithms in the field vary significantly by the EV-specific features considered, the complexity of the methodology and application use cases. The first group of algorithms uses detailed energy consumption models respecting the EV’s ability to recuperate energy. Concurrently, these algorithms neglect the possibility of battery recharges on the way. Researchers in (Cauwer et al., 2019) used the shortest path algorithm to find the optimal energy route on a weighted graph with a data-driven prediction of energy consumption. Authors in (Abousleiman and Rawashdeh, 2014) deployed the ant colony and particle swarm optimization to generate the most energy-efficient route. Despite being fast, the solution is tedious to formulate and requires adaptation to different EV usage cases. An interesting approach based on learning from historical driving data is demonstrated in (Bozorgi et al., 2017). The proposed solution aims at minimizing both energy consumption and travel time while accommodating particular driving habits. The second group of algorithms focuses on EV’s interaction with charging stations while considering constant energy consumption without energy recuperation. (Sweda and Klabjan, 2012) used approximate dynamic programming to minimize traveling and recharging costs. (Daanish and Naick, 2017) deployed a nearest neighbour search-based algorithm to find the shortest energy-efficient path. Researchers in (Schoenberg and Dressler, 2019) and (Tang et al., 2019) proposed algorithms to reduce the total travel time. The prior suggested a multi-criterion shortest path search with an adaptive charging strategy. The latter solved a joint routing and charging scheduling optimization problem that additionally minimizes the monetary cost. The third group demonstrates an improvement in EV routing by considering both energy recuperation and battery recharging. A dynamic programming approach was proposed in (Pourazarm et al., 2014) to minimize total travel time in the road network defined as a graph. Despite successful application for a case of one car, the approach showed poor scalability in terms of convergence speed when the number of vehicles increased. (Morlock et al., 2019) suggested a trip planner that solves a mixed integer linear program to reduce the overall trip time. The authors introduced the driving speed as an additional degree of freedom and forecasted energy consumption from historical data. However, their approach works only along the desired route without considering alternative trajectories.

Although the majority of the proposed algorithms deal with route planning for casual EV driving, the efforts are made to adapt EVs for specific use cases of customer serving and delivery operations. Researchers in (Schneider et al., 2014) deployed a hybrid heuristic search algorithm to minimize the total time consisting of travel time, recharging time and time spent at the customer. Authors in (Mao et al., 2020) aimed for the same goal with battery swapping and fast charging options using improved ant colony optimization. (Felipe et al., 2014) used simulated annealing to find a feasible route while determining the amount of energy to be recharged at the charging station along with the type of charging technology. Despite considering the recharging possibilities on the way, these works neglect the EV’s ability to recuperate energy by assuming constant energy consumption proportional to the travel distance.

This paper aims to address highlighted drawbacks in the EV-specific route planning by proposing a novel problem formulation suitable for solving by reinforcement learning (RL) techniques. To the best of our knowledge, it is one of the first applications of this area of machine learning to the field of EV path planning. Previously, the success of using RL, namely the policy gradient algorithm, was demonstrated in (Nazari et al., 2018) to minimize the total route length of a conventional fossil-fuel vehicle. Additionally, researchers in (Zhang Q. et al., 2020) used actor-critic RL to minimize the route’s energy consumption without recharging opportunities. In (Zhang C. et al., 2020), a deep RL approach was proposed to reduce both travel time and distance while different charging modes and occupation of charging spots were considered. In this research, we formulate the EV-specific routing problem in a graph-theoretical setting as a Markov decision process (MDP) and suggest a possible model-free RL algorithm to solve it by generating energy feasible paths for EV from source to target. Specifically, we take into account recharging possibilities on the way through intermediary charging stations and the ability of EV to recuperate energy by considering the elevation profile of the road network.

## 2 Method

Two main components are required to frame the problem of EV routing with intermediary charging stations. First, the environment where an EV operates, namely the road network, has to be described mathematically. In this research, EV routing is analyzed in a graph-theoretical context. Second, the problem has to be formulated as an MDP to provide modelling capabilities of the EV movement and its way of making decisions.

### 2.1 Environment

The road network can be modelled as a simple undirected weighted graph G=(V,E) as follows:

• V={1,…,n} is the set of *n* nodes representing the points of interest on the map. The subset of these nodes C={1,…,m}⊂V can provide recharging capabilities to EVs. Each of the nodes $v_{i}\in V$ can serve both as a source $v_{0}$ and as a target $v_{f}$ that are EV’s starting and destination points respectively. To consider the EVs’ ability to recuperate energy when moving downhill, we characterize each node $v_{i}\in V$ by its elevation $z_{i}$.
• E⊂ℝ is the set of weighted edges that connect the nodes on the graph. Each edge can be defined as an unordered pair $\left\{v_{i},v_{j}\right\}$, where $v_{i}\neq v_{j}$. There are no multiple edges that are incident to the same two nodes. As the graph *G* is undirected, the edges are equivalent to two-way roads in the real world. The weights of the edges correspond to the energy costs required to traverse the edge.

The definition of edges’ weights was adapted from (Bozorgi et al., 2017). Therefore, the energy cost between two nodes $v_{i}$ and $v_{j}$ can be determined as follows:

$$E_{ij}=E_{flat_{ij}}+E_{inclined_{ij}}+E_{other_{ij}}$$ (1)

where $E_{flat_{ij}}$ and $E_{inclined_{ij}}$ represent EV’s energy consumption on flat and inclined surfaces respectively. The term $E_{other_{ij}}$ signifies additional energy costs depending on road type, urbanization, weather conditions and usage of auxiliary components (Li et al., 2016). For the sake of simplicity, $E_{other_{ij}}=0$. The basic energy consumption on the flat road can be determined according to Equation 2, where *h* is the EV’s specific energy consumption per 100 km and $d_{ij}$ is the distance between nodes. The value of *h* is determined experimentally for different models of EVs according to typical driving cycles such as WLTP (European Automobile Manufacturers Association, 2017).

$$E_{flat_{ij}}=d_{ij}h$$ (2)

The contribution of an inclined surface to EV’s energy consumption is proportional to the potential energy and can be calculated as follows:

$$E_{inclined_{ij}}=mg\text{Δ}z/\eta$$ (3)

where *m* is the combined mass of EV and its payload, *g* is the acceleration of gravity, $\text{Δ}z=z_{j}-z_{i}$ is the elevation difference between nodes, and *η* is the EV’s transmission efficiency. The value of $E_{inclined_{ij}}$ is responsible for EV’s energy recuperation ability. In downhill, Δz<0, therefore $E_{inclined_{ij}}<0$ and EV can recuperate energy if $|E_{inclined_{ij}}|\;\;\;>\;\;E_{flat_{ij}}$. In contrast, Δz>0 when EV moves uphill, thus $E_{inclined_{ij}}>0$ and additional energy has to be spent. If two nodes have no edge connecting them, the weight $E_{ij}=\infty$ makes it impossible for EV to traverse the graph in this direction.

### 2.2 Markov Decision Process

**FIGURE 1:**
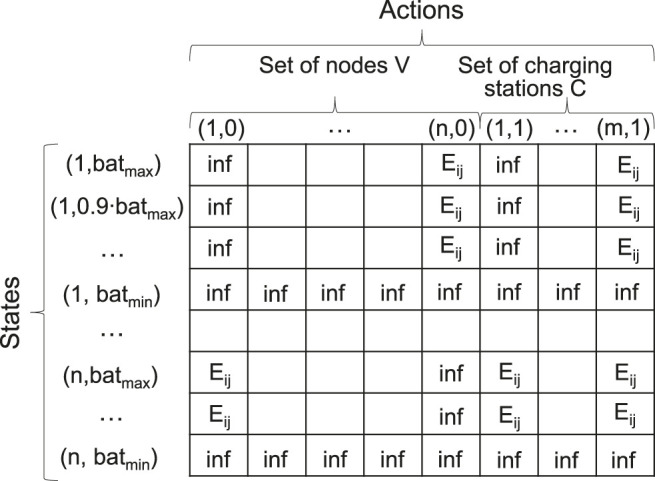
Matrix representation of a combined state-action space.

To formulate the EV-specific routing problem, we use an MDP mathematical framework which provides the best way to generalize optimal behaviour problems under uncertainty. An MDP model (S,A,P,R,γ) consists of the following elements: a finite set of states *S*, where each of them obeys the Markovian property, a finite set of actions *A*, state transition probability matrix *P*, rewards function *R*, and discount factor *γ*. The definition of states and actions is related to the graph-theoretical context of the problem and can be represented as a matrix depicted in Figure 1.

State space *S* contains all possible states *s* that an agent can have when interacting with a given environment. For the case of EV routing, a state can be described as a vector *s = (location, charge)*, where location∈V and *charge* corresponds to the battery energy level. The latter is constrained due to battery’s operational limits such as *bat*_*min*_ *≤ charge ≤ bat*_*max*_. The upper bound *bat*_*max*_ is imposed by the battery capacity and the lower bound *bat*_*min*_ is determined by the advised discharging policy. As most rechargeable batteries are not meant to be fully discharged, a minimum allowed state of charge is set to avoid battery damage. In this research we assume *bat*_*min*_ *= 20%bat*_*max*_. Contrary to *location*, *charge* is a continuous variable requiring discretization that can be achieved through binning. The number of bins is determined experimentally through uniform binning, where the bin’s lower bound defines the new state, once the action is executed. The discretization procedure is discussed further in Section 4.1.

Action space *A* contains all possible actions that an agent can perform in the environment. An action can be described as a vector *a = (next_location, decision)*, where next_location∈V and decision indicates the charging intention at this location. If next_location∈C, an agent can choose whether to charge *decision = 1* at this node or not *decision = 0*. If next_location∉C, the agent has no choice and *decision = 0*. However, at any state *s* not all actions are available to the agent. The action *a* is considered available at state *s* only if $charge_{s}-E_{sa}\geq bat_{min}$, where $E_{sa}$ is the energy cost to move from *location* to *next_location*.

Rewards function *R* is a measure to encourage the particular behaviour of an agent. While interacting with the environment, the agent takes action from the current state, observes the new state and receives a reward. By continually getting feedback from the environment in the form of rewards, the agent learns the desired behaviour through maximizing its discounted cumulative reward. In the EV-specific routing problem, we mainly want to incentivize only one type of behaviour by setting reward equal to 1: reaching the target $v_{f}$ from the source $v_{0}$ with charge level *charge ≥ bat*_*min*_. Rewarding the arrival to the final destination is essential for the agent’s understanding that it has to explore the graph in a specific direction and not just wander around the environment. However, not all rewards have to be positive. Sometimes, rewards are used to penalize particular behaviour. In the current case of EV routing, an agent receives a negative reward equal to −1 when there are no available actions at the current state. In the real world, it means that EV has exhausted its battery capacity and thus got stuck on its route before reaching the destination.

Discount factor *γ* is used to emphasize the importance of the rewards achieved in the future. The agent selects actions to maximize the cumulative discounted reward *G*_*t*_ at time point *t* according to Equation 4, where *R*_*t*_ signifies the reward’s value at time *t* and *n* defines the number of steps to complete the task. The discount rate *γ* obeys 0≤γ≤1, therefore one needs to find balance between caring about immediate rewards only (γ=0) and caring about distant future (γ=1).

$$G_{t}=R_{t+1}+\gamma R_{t+2}+\gamma^{2}R_{t+3}+\gamma^{3}R_{t+4}+\ldots +\gamma^{n-1}R_{t+n}$$ (4)

In this research, we do not calculate explicitly the state transition probability matrix *P* due to the following assumptions in formulating the EV-specific routing problem. First, we do not consider specific traffic conditions. It is common for drivers to plan their routes according to traffic congestion and even change them while driving. Therefore, the probability of choosing a particular road would need to be adjusted dynamically. Second, as we aim to solve the routing problem for energy feasibility, we do not take into account the occupation of the charging stations and the time required for charging. Third, we assume that there are no partial recharges and that all EVs leave the charging station with the full battery. Moreover, although the behaviour of an EV driver is presumed to be rational, in the real world, it is still stochastic. The drivers are free to choose the next points on their path according to any unforeseen events or their personal beliefs. Considering all the points discussed above, calculating the state transition probability matrix *P* that would accurately reflect real-world environment dynamics does not seem possible. Therefore, a model-free RL algorithm that operates regardless of any representation of *P* should be selected to solve the suggested MDP. To find the target policy that fully defines the agent’s desired behavior, we deploy the off-policy learning method that allows to do it independently from the followed exploratory policy.

### 2.3 Algorithm

**FIGURE 2:**
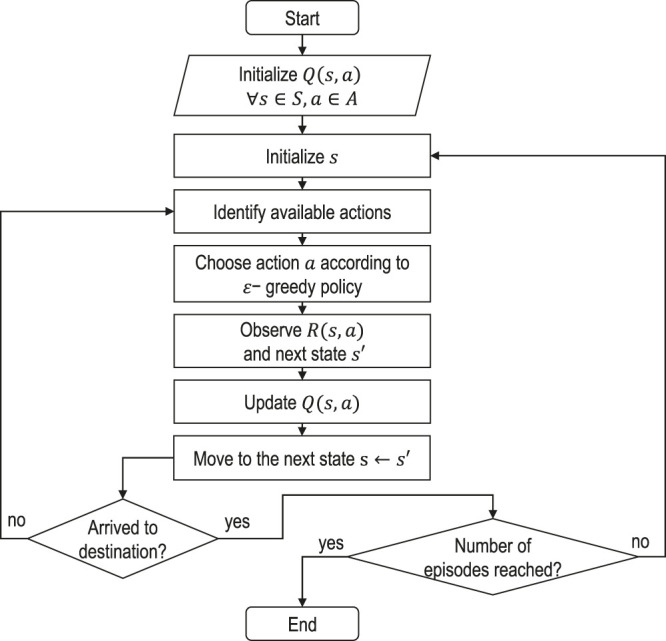
One-step ahead Q-learning algorithm.

As one of the possible methods to solve the suggested MDP formulation of the EV-specific routing problem, we choose the Q-learning algorithm, which is a specific instance of temporal difference learning that looks only one step ahead. Moreover, it is suitable for discrete state and action spaces and is easily interpretable. The idea of Q-learning is to allow improvements for both target and exploratory policies. The target policy is a greedy policy that obeys the following definition:

$$\pi \left(s^{\prime }\right)=\underset{a^{\prime }}{\text{arg}\;\text{max}}\;Q\left(s^{\prime },a^{\prime }\right)$$ (5)

where *π* is the policy, *Q* is the action-value function, $s^{\prime }$ is the next state and $a^{\prime }$ is some alternative action that maximizes the Q-value. The real behavioural policy that the agent follows is an *ϵ*-greedy policy which ensures continual exploration. The policy is defined as follows:

$$\pi \left(a\;|\;s\right)=\{\begin{array}{ll} \epsilon /m+1-\text{ɛ},if\;a^{\text{*}}=\underset{a\in A}{\text{arg}\;\text{max}}\;Q\left(s,a\right) & \\ \epsilon /m,\;\;otherwise & \end{array}$$ (6)

where *s* and *a* are the current state and action taken at this state, *ϵ* is a parameter that governs the exploration-exploitation trade-off, *m* is the number of actions available at the current state, and a^*^ is the best possible action. The Q-value function is updated according to Bellman’s optimality equation in the following way:

$$Q\left(s,a\right)\;\leftarrow \;Q\left(s,a\right)+\alpha \;\left[R\left(s,a\right)+\gamma \;\underset{a^{\prime }}{\text{max}}\;Q\left(s^{\prime },a^{\prime }\right)-Q\left(s,a\right)\right]$$ (7)

where Q(s,a) is the Q-value of the current state and action pair, R(s,a) is the observed reward after the action *a* is taken and *α* is the learning rate bounded by 0≤α≤1. The latter determines to what extent newly acquired information overrides old information. The complete Q-learning algorithm is described in Figure 2.

## 3 Results

### 3.1 Case Study

**FIGURE 3:**
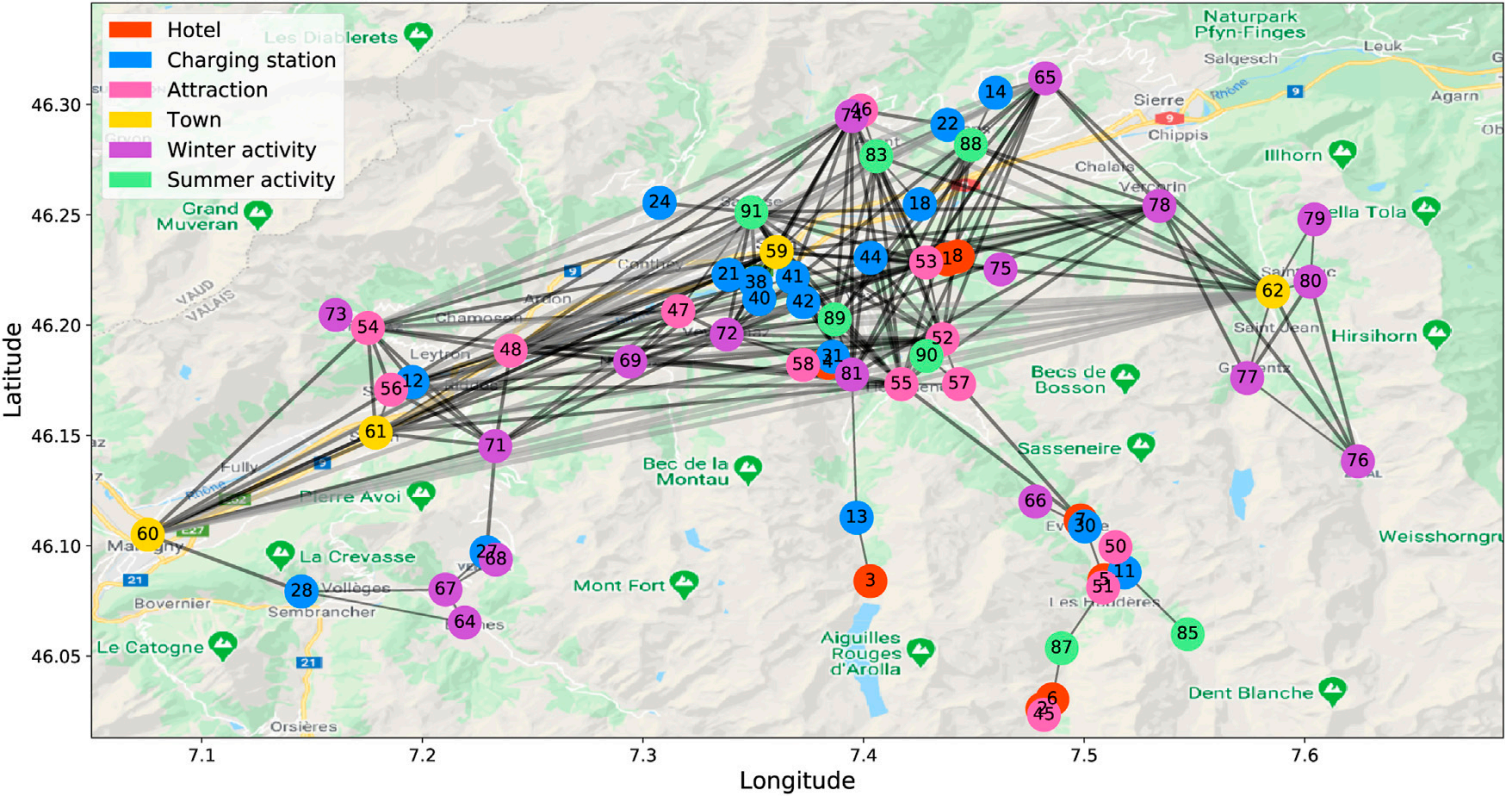
Graph representation of the road network of the Val d’Hérens alpine region in Switzerland.

To validate the proposed method for solving the EV-specific routing problem, we created a case study within the framework of the Digitalization project (SCCER, 2020). The case study deals with the section of the road network of the Val d’Hérens alpine region in Switzerland. Figure 3 depicts the graph representation of the road network. The environment encompasses 66 nodes and 223 edges, which represent the points of interest and the connection roads, respectively. The thickness of the edges varies depending on the relative remoteness of the nodes. Each node is characterized by its geographical coordinates: latitude, longitude, and elevation. The agent is an EV defined by its battery capacity, energy consumption rate, and mass. In our case study, we use Citroen C-Zero with 16 kWh battery and an average 12.6 kWh energy consumption per 100 km (Electric vehicle database, 2020).

### 3.2 Training

The training procedure in RL is defined as a sequence of episodes. One episode represents the movement of an agent along the path from source to target. The episode is considered complete when the target is reached. The number of episodes should be sufficient to achieve a stable matrix of Q-values which is initialized to zeros at the beginning of the training procedure. Such Q-matrix represents the maximum expected future rewards for each action at each state. The training convergence is achieved when the updates of the old Q-values become insignificant. Therefore, an agent learns the optimal policy once the algorithm converges. The parameters that govern the training process are set to the following values: discount factor γ=0.9, learning rate α=0.8, and ϵ=0.1. The values are tuned experimentally to ensure convergence and satisfactory execution speed.

**FIGURE 4:**
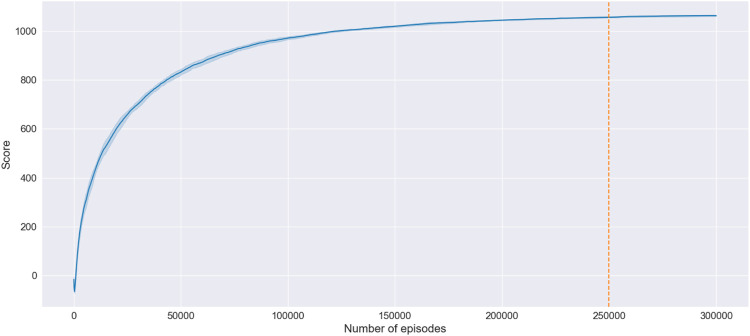
An example of a learning curve of the training process. The bold line and the shaded region show the mean and the standard deviation of five runs.

Figure 4 depicts an example of a learning curve of the algorithm’s training process, where the x-axis denotes the number of episodes, while the y-axis represents the training score. The episode’s training score is determined by the mean of the scores obtained at each step of the episode. The step’s score is calculated as a sum of the Q-values in the Q-matrix. Therefore, the learning curve arrives at a plateau when the Q-matrix stabilizes.

In the demonstrated example, the algorithm converges after 250,000 episodes, which takes approximately 6.2 min. The Q-learning was programmed in Python, and the training procedure was executed on a personal laptop (Intel i7- 7600, 16 GB RAM). One has to note that training uses a fixed target while the source is chosen arbitrarily. Therefore, the algorithm requires retraining when the destination is changed. Notably, any topological modifications of the road network, such as introducing additional nodes or removing existing ones, would equally require retraining of the algorithm.

### 3.3 Validation

A series of experiments, where each node sequentially serves as a target, is carried out iteratively to test the consistency of the policy learned by the agent with the energy feasibility goal. Each experiment simulates an EV trip starting at a random node on the graph with the fully charged battery and finishing when the final destination is reached. For each target, the amount of experiments equals *N*−1, where *N* = 66 is the number of nodes in the selected road network. Thus, the total number of experiments is 4,290. Besides verifying the EV’s capability of arriving at the target without violating the *bat*_*min*_ constraint, we aim to observe whether the EV stops to recharge only when it is strictly necessary. Although not accounted for in the reward function’s design, excessive charging behavior is not preferable. Thus, observing the frequency of unnecessary charging stops contributes to further improving the solution.

The results demonstrate that 100% of generated routes are energy feasible, while 92% of them represent near-optimal charging decisions. The latter means that recharging schemes suggested by the algorithm give the agent a possibility to arrive at the destination, otherwise unreachable without charging, and neglect to charge when it is attainable to arrive at the destination without violating battery constraints. Moreover, the results show that in 80% of cases, the optimal number of charging stops was selected, thus avoiding excessive charging. Such a number is calculated using a verification procedure that analyzes the route with all possible combinations of the charging stations proposed by the algorithm. Although we did not aim to optimize for the route length, an interesting observation occurred. In 83% of cases, the algorithm generated the shortest possible path when recharging is not required, which was confirmed by the Dijkstra algorithm. To summarize, we validated the possible use of a Q-learning algorithm to solve the proposed formulation of the EV-specific routing problem. The following section discusses the advantages and limitations of the suggested approach and defines the directions for future research.

## 4 Discussion

The MDP formulation of the EV-specific routing problem and the proposed model-free RL approach have certain advantages in comparison to previous works in the literature. First, our method considers both main properties of EVs: a possibility to recharge on the way and an energy recuperation ability. Although these features are crucial to model the agent’s behaviour that will be close to real-world driving habits, taking into account both of them is uncommon, as shown in Section 1. Moreover, compared to previous RL works, the prior was neglected in (Zhang Q. et al., 2020). The latter was considered in (Zhang C. et al., 2020) through estimating energy consumption from rarely available historical data. Second, a trained RL agent requires less computing effort and less memory space than model-based techniques and mixed integer non-linear programming formulations (Mocanu et al., 2018) of the EV routing problem such as (Pourazarm et al., 2014). Thus, it can be deployed for online applications if successfully transferred to the real world. Third, problem formulation in a graph setting and usage of the Q-learning algorithm that employs Q-matrix make results’ interpretation more intuitive. Last but not least, the off-policy temporal difference continuously evaluates the returns from the environment and makes incremental updates using bootstrapping. Therefore, unlike the Monte-Carlo approach, it is not necessary to wait until the episode terminates to judge the agent’s behaviour.

### 4.1 Limitations

Although the suggested approach has some inherent advantages, it also has certain limitations influencing performance. The first limitation comes from the choice of the algorithm. The Q-learning is suitable for problems with small to medium size of a state-action space as it stores information in the form of Q-tables. Once the dimensions of the problem increase, the algorithm scales poorly. In the proposed framework, the growth of a state-action space can come from the expansion of the road network and the state discretization procedure. The selected binning method represents a simple way to discretize a continuous *battery* variable, where the number of bins is chosen as a trade-off between the level of detail at which we model the problem and the size of the state space. With a large number of states and actions, the probability of visiting a particular state and performing specific action decreases dramatically, thus deteriorating the performance, slowing down the training process, and exhibiting higher memory demands. To solve the scaling issue, one can use function approximators, such as neural networks or tile coding, or switch to policy-based RL. The second limitation comes from fixing the minimum required battery charge at the target $v_{f}$ to *bat*_*min*_. As some destinations might not have charging stations, the EVs can get stuck without sufficient battery charge to start a new trip. Therefore, one has to introduce an additional parameter *bat*_*f*_ that depends on *v*_*f*_ and ensures that the battery charge at the destination is sufficient to arrive at the closest charging station. The third limitation of the method’s applicability is the need to retrain the algorithm when the destination is changed or any topological modifications occur to the road network. Thus, it should be clearly addressed to improve the method’s convenience for end-users. Finally, the agent’s evaluation on the same environment model used for training questions its real-world performance and the ability to handle stochastic perturbations.

### 4.2 Future Work

The assumptions made in formulating the EV-specific routing problem define the directions for future improvements. First, the goal of the learning process can be tailored according to the desired application by altering the rewards scheme. One can diversify the routing problem towards minimizing travel time, travel distance, total energy consumption, and the number of recharging stops. Second, specific characteristics of the charging process, such as charging time and charging intensity, can be considered. Moreover, one can differentiate charging stations by their slot availability and suggested price of electricity, thus introducing additional decision variables. Another improvement can be realized by including partial recharges. Therefore, the agent will have to choose not only the charging station but the amount of recharge too. Third, one can consider dynamic traffic conditions to build an environment that resembles the real world. Inclusion of traffic will affect the actions’ availability and the agent’s energy consumption model. The latter can be improved by accounting for the type of terrain, use of auxiliary loads, and weather conditions. Fourth, the suggested approach to EV-specific routing can be extended towards the multi-agent RL problem. Although this area of artificial intelligence is still in its infancy, the attempts to modelling road networks with multiple agents can foster developments in the field and can help to build improved foundations for autonomous green mobility. Finally, one should devote the efforts to benchmark the suggested methodology against other popular approaches for solving the routing problem. Moreover, further investigation of the agent’s validity in the real world, beyond simulations, is required, preferably supported by experimental results in practice.

## 5 Conclusion

In this work, we proposed a mathematical formulation of the EV-specific routing problem, and we demonstrated a possible solution using a model-free RL approach. We defined the problem as an incomplete MDP in a graph-theoretical context. To generate energy feasible paths, we implemented an off-policy temporal difference algorithm with one step ahead. Notably, our framework considers recharging possibilities at intermediary charging stations and the ability of EVs to recuperate energy. We demonstrated in a case study that the algorithm always produces energy feasible paths. The training procedure of the algorithm requires low computational and memory demands and is suitable for online applications.

## Data Availability

The raw data supporting the conclusion of this article will be made available by the authors, without undue reservation.
